# Supplementary material for: Mutational Landscape and Actionable Target Rates on Advanced Stage Refractory Cancer Patients: A Multicenter Chilean Experience
Source: J Pers Med. 2022 Jan 31;12(2):195. doi: 10.3390/jpm12020195 (PMC8879850; doi:10.3390/jpm12020195)
Supplement: Supplementary file 1 [file jpm-12-00195-s001.zip › Supplementary Table S1.pdf]

Supplementary Table S1. Main characteristic of platforms used for tumor profiling

**Platform**	**# of genes analyzed**	**NGS technology**	**SNV and CNA**	**Fusion**	**Tumor tissue or liquid biopsy seq**
OncoDeep	313	Amplicon Seq	Yes	No	Tumor
OncoSelect	15	Amplicon Seq	Yes	Yes	Liquid biopsy
OncoSTRAT	~200	Amplicon Seq	Yes	Yes	Tumor and liquid biopsy
OncoDeep DX+	65	Amplicon Seq	Yes	No	Tumor
BGI SENTIS Cancer+Discovery Tissue	688	Hybridization capture	Yes	Yes	Tumor
BGI SENTIS Cancer+Discovery ctDNA	688	Hybridization capture	Yes	Yes	Liquid biopsy
Admera oncoGxone	364	Hybridization capture	Yes	Yes	Tumor
Caris Comprehensive Tumor Profiling	592	Hybridization capture	Yes	Yes	Tumor
FoundationOne CDx	324	Hybridization capture	Yes	Yes	Tumor
FoundationOne Heme	406	Hybridization capture	Yes	Yes	Tumor
FoundationOne liquid	70	Hybridization capture	Yes	Yes	Liquid biopsy
FoundationOne	315	Hybridization capture	Yes	Yes	Tumor
MD Anderson Madrid	161	Amplicon Seq	Yes	Yes	Tumor

NGS Next-Generation Sequencing, Seq sequencing, SNV Single Nucleotide Variant, CNA Copy Number Alteration.
