# Supplementary material for: Mutational Landscape and Actionable Target Rates on Advanced Stage Refractory Cancer Patients: A Multicenter Chilean Experience
Source: J Pers Med. 2022 Jan 31;12(2):195. doi: 10.3390/jpm12020195 (PMC8879850; doi:10.3390/jpm12020195)
Supplement: Supplementary file 1 [file jpm-12-00195-s001.zip › Supplementary Table S2.pdf]

Supplementary Table S2. Platforms used for tumor molecular profile according to cancer type.

ID	Cancer type	Platform tumor profile	Tumor tissue or liquid biopsy sample
1	Breast cancer	OncoDeep	Tumor tissue sample
2	Kidney cancer	OncoDeep	Tumor tissue sample
3	Unknown primary	OncoDeep	Tumor tissue sample
4	Esophageal cancer	OncoDeep	Tumor tissue sample
5	Kidney cancer	OncoDeep	Tumor tissue sample
6	Pancreatic cancer	OncoDeep	Tumor tissue sample
7	Breast cancer	OncoDeep	Tumor tissue sample
8	Central nervous system	OncoDeep	Tumor tissue sample
9	Pancreatic cancer	OncoDeep	Tumor tissue sample
10	Breast cancer	OncoDeep	Tumor tissue sample
11	Ovarian cancer	OncoDeep	Tumor tissue sample
12	Uterine cancer	OncoDeep	Tumor tissue sample
13	Colorectal cancer	OncoDeep	Tumor tissue sample
14	Unknown primary	OncoDeep	Tumor tissue sample
15	Colorectal cancer	OncoDeep	Tumor tissue sample
16	Bladder cancer	OncoDeep	Tumor tissue sample
17	Hepatic cancer	OncoDeep	Tumor tissue sample
18	Esophageal cancer	OncoDeep	Tumor tissue sample
19	Sarcoma	OncoDeep	Tumor tissue sample
20	Breast cancer	OncoDeep	Tumor tissue sample
21	Lung cancer	OncoDeep	Tumor tissue sample
22	Esophageal cancer	OncoDeep	Tumor tissue sample
23	Sarcoma	OncoDeep	Tumor tissue sample
24	Pancreatic cancer	OncoDeep	Tumor tissue sample
25	Colorectal cancer	OncoDeep	Tumor tissue sample
26	Thymic cancer	OncoDeep	Tumor tissue sample
27	Colorectal cancer	OncoDeep	Tumor tissue sample
28	Ovarian cancer	OncoDeep	Tumor tissue sample
29	Kidney cancer	OncoDeep	Tumor tissue sample
30	Colorectal cancer	OncoDeep	Tumor tissue sample
31	Central nervous system	OncoDeep	Tumor tissue sample
32	Colorectal cancer	OncoDeep	Tumor tissue sample
33	Lung cancer	OncoDeep	Tumor tissue sample
34	Colorectal cancer	OncoDeep	Tumor tissue sample
35	Colorectal cancer	OncoDeep	Tumor tissue sample
36	Pancreatic cancer	OncoDeep	Tumor tissue sample

37	Gastric cancer	OncoDeep	Tumor tissue sample
38	Lung cancer	OncoDeep	Tumor tissue sample
39	Gallbladder/bile duct	OncoDeep	Tumor tissue sample
40	Pancreatic cancer	OncoDeep	Tumor tissue sample
41	Colorectal cancer	OncoDeep	Tumor tissue sample
42	Gallbladder/bile duct	OncoDeep	Tumor tissue sample
43	Gallbladder/bile duct	OncoDeep	Tumor tissue sample
44	Pancreatic cancer	OncoDeep	Tumor tissue sample
45	Sarcoma	OncoDeep	Tumor tissue sample
46	Pancreatic cancer	OncoDeep	Tumor tissue sample
47	Gallbladder/bile duct	OncoDeep	Tumor tissue sample
48	Colorectal cancer	OncoDeep	Tumor tissue sample
49	Gallbladder/bile duct	OncoSTRAT	Tumor and liquid biopsy sample
50	Unknown primary	OncoDeep	Tumor tissue sample
51	Colorectal cancer	OncoDeep	Tumor tissue sample
52	Colorectal cancer	OncoSTRAT	Tumor and liquid biopsy sample
53	Colorectal cancer	OncoDeep	Tumor tissue sample
54	Sarcoma	OncoDeep	Tumor tissue sample
55	Lung cancer	OncoSelect	Liquid biopsy sample
56	Lung cancer	OncoDeep	Tumor tissue sample
57	Kidney cancer	OncoDeep	Tumor tissue sample
58	Pancreatic cancer	OncoDeep	Tumor tissue sample
59	Breast cancer	OncoDeep	Tumor tissue sample
60	Bladder cancer	OncoDeep	Tumor tissue sample
61	Gallbladder/bile duct	OncoDeep	Tumor tissue sample
62	Pancreatic cancer	OncoDeep	Tumor tissue sample
63	Colorectal cancer	OncoSTRAT	Tumor and liquid biopsy sample
64	Pancreatic cancer	OncoDeep	Tumor tissue sample
65	Sarcoma	OncoDeep	Tumor tissue sample
66	Ovarian cancer	OncoDeep	Tumor tissue sample
67	Kidney cancer	OncoDeep	Tumor tissue sample
68	Unknown primary	OncoDeep	Tumor tissue sample
69	Bladder cancer	OncoDeep	Tumor tissue sample
70	Sarcoma	OncoDeep	Tumor tissue sample
71	Ovarian cancer	OncoDeep	Tumor tissue sample
72	Ovarian cancer	OncoDeep	Tumor tissue sample
73	Colorectal cancer	OncoDeep	Tumor tissue sample
74	Prostate cancer	OncoDeep	Tumor tissue sample
75	Uterine cancer	OncoDeep	Tumor tissue sample

76	Uterine cancer	OncoDeep	Tumor tissue sample
77	Nonmelanoma/skin	OncoDeep	Tumor tissue sample
78	Colorectal cancer	OncoDeep	Tumor tissue sample
79	Gastric cancer	OncoDeep	Tumor tissue sample
80	Sarcoma	OncoDeep	Tumor tissue sample
81	Pancreatic cancer	OncoDeep	Tumor tissue sample
82	Sarcoma	OncoDeep	Tumor tissue sample
83	Neuroendocrine tumor	OncoDeep	Tumor tissue sample
84	Sarcoma	OncoDeep	Tumor tissue sample
85	Ovarian cancer	OncoDeep	Tumor tissue sample
86	Adrenal cancer	OncoDeep	Tumor tissue sample
87	Ovarian cancer	OncoDeep	Tumor tissue sample
88	Unknown primary	OncoDeep	Tumor tissue sample
89	Colorectal cancer	OncoDeep	Tumor tissue sample
90	Colorectal cancer	OncoDeep	Tumor tissue sample
91	Breast cancer	OncoDeep	Tumor tissue sample
92	Unknown primary	OncoDeep	Tumor tissue sample
93	Hepatic cancer	OncoDeep	Tumor tissue sample
94	Central nervous system	OncoDeep	Tumor tissue sample
95	Sarcoma	OncoDeep	Tumor tissue sample
96	Unknown primary	OncoDeep	Tumor tissue sample
97	Breast cancer	OncoDeep	Tumor tissue sample
98	Gallbladder/bile duct	OncoDeep	Tumor tissue sample
99	Ovarian cancer	OncoDeep	Tumor tissue sample
100	Colorectal cancer	OncoDeep	Tumor tissue sample
101	Uterine cancer	OncoDeep DX+	Tumor tissue sample
102	Sarcoma	OncoDeep DX+	Tumor tissue sample
103	Pancreatic cancer	OncoDeep DX+	Tumor tissue sample
104	Colorectal cancer	OncoDeep DX+	Tumor tissue sample
105	Kidney cancer	OncoDeep DX+	Tumor tissue sample
106	Kidney cancer	OncoDeep DX+	Tumor tissue sample
107	Central nervous system	OncoDeep DX+	Tumor tissue sample
108	Central nervous system	OncoDeep DX+	Tumor tissue sample
109	Gallbladder/bile duct	OncoDeep DX+	Tumor tissue sample
110	Breast cancer	OncoDeep DX+	Tumor tissue sample
111	Pancreatic cancer	OncoDeep DX+	Tumor tissue sample
112	Breast cancer	OncoDeep DX+	Tumor tissue sample
113	Testicular cancer	OncoDeep DX+	Tumor tissue sample
114	Breast cancer	OncoDeep DX+	Tumor tissue sample

115	Gastric cancer	OncoDeep DX+	Tumor tissue sample
116	Colorectal cancer	OncoDeep DX+	Tumor tissue sample
117	Sarcoma	OncoDeep DX+	Tumor tissue sample
118	Primary peritoneal cancer	OncoDeep DX+	Tumor tissue sample
119	Kidney cancer	OncoDeep DX+	Tumor tissue sample
120	Sarcoma	OncoDeep DX+	Tumor tissue sample
121	Lung cancer	OncoDeep DX+	Tumor tissue sample
122	Breast cancer	OncoDeep DX+	Tumor tissue sample
123	Central nervous system	OncoDeep DX+	Tumor tissue sample
124	Testicular cancer	OncoDeep DX+	Tumor tissue sample
125	Pancreatic cancer	OncoDeep DX+	Tumor tissue sample
126	Pancreatic cancer	OncoDeep DX+	Tumor tissue sample
127	Unknown primary	OncoDeep DX+	Tumor tissue sample
128	Esophageal cancer	OncoDeep DX+	Tumor tissue sample
129	Bladder cancer	OncoDeep DX+	Tumor tissue sample
130	Unknown primary	OncoDeep DX+	Tumor tissue sample
131	Lung cancer	OncoDeep DX+	Tumor tissue sample
132	Breast cancer	OncoDeep DX+	Tumor tissue sample
133	Lung cancer	OncoDeep DX+	Tumor tissue sample
134	Gastric cancer	OncoDeep DX+	Tumor tissue sample
135	Lung cancer	OncoDeep DX+	Tumor tissue sample
136	Colorectal cancer	OncoDeep DX+	Tumor tissue sample
137	Lung cancer	OncoDeep DX+	Tumor tissue sample
138	Lung cancer	OncoDeep DX+	Tumor tissue sample
139	Breast cancer	OncoDeep DX+	Tumor tissue sample
140	Colorectal cancer	OncoDeep DX+	Tumor tissue sample
141	Salivary gland cancer	OncoDeep DX+	Tumor tissue sample
142	Colorectal cancer	OncoDeep DX+	Tumor tissue sample
143	Colorectal cancer	OncoDeep DX+	Tumor tissue sample
144	Sarcoma	OncoDeep DX+	Tumor tissue sample
145	Pancreatic cancer	OncoDeep DX+	Tumor tissue sample
146	Colorectal cancer	OncoDeep DX+	Tumor tissue sample
147	Unknown primary	OncoDeep DX+	Tumor tissue sample
148	Lung cancer	OncoDeep DX+	Tumor tissue sample
149	Pancreatic cancer	OncoDeep DX+	Tumor tissue sample
150	Sarcoma	OncoDeep DX+	Tumor tissue sample
151	Unknown primary	OncoDeep DX+	Tumor tissue sample
152	Unknown primary	OncoDeep DX+	Tumor tissue sample
153	Unknown primary	OncoDeep DX+	Tumor tissue sample

154	Colorectal cancer	OncoDeep DX+	Tumor tissue sample
155	Unknown primary	OncoDeep DX+	Tumor tissue sample
156	Ovarian cancer	OncoDeep	Tumor tissue sample
MG1	Pancreatic cancer	FoundationOne	Tumor tissue sample
MG2	Neuroendocrine tumor	BGI SENTIS Cancer+Discovery ctDNA	Liquid biopsy sample
MG3	Colorectal cancer	FoundationOne CDx	Tumor tissue sample
MG4	Pancreatic cancer	BGI SENTIS Cancer+Discovery ctDNA	Liquid biopsy sample
MG5	Ovarian cancer	FoundationOne CDx	Tumor tissue sample
MG6	Colorectal cancer	BGI SENTIS Cancer+Discovery Tissue	Tumor tissue sample
MG7	Colorectal cancer	BGI SENTIS Cancer+Discovery Tissue	Tumor tissue sample
MG8	Gallbladder/bile duct	BGI SENTIS Cancer+Discovery ctDNA	Liquid biopsy sample
MG9	Colorectal cancer	BGI SENTIS Cancer+Discovery ctDNA	Liquid biopsy sample
MG10	Colorectal cancer	BGI SENTIS Cancer+Discovery ctDNA	Liquid biopsy sample
MG11	Melanoma	BGI SENTIS Cancer+Discovery ctDNA	Liquid biopsy sample
MG12	Colorectal cancer	BGI SENTIS Cancer+Discovery ctDNA	Liquid biopsy sample
MG13	Neuroendocrine tumor	BGI SENTIS Cancer+Discovery Tissue	Tumor tissue sample
MG14	GIST	BGI SENTIS Cancer+Discovery Tissue	Tumor tissue sample
MG15	Adrenal cancer	BGI SENTIS Cancer+Discovery Tissue	Tumor tissue sample
MG16	Adrenal cancer	BGI SENTIS Cancer+Discovery Tissue	Tumor tissue sample
MG17	Colorectal cancer	BGI SENTIS Cancer+Discovery ctDNA	Liquid biopsy sample
MG18	GIST	BGI SENTIS Cancer+Discovery ctDNA	Liquid biopsy sample
MG19	Prostate cancer	BGI SENTIS Cancer+Discovery ctDNA	Liquid biopsy sample
MG20	Pancreatic cancer	FoundationOne CDx	Tumor tissue sample
MG21	Esophageal cancer	BGI SENTIS Cancer+Discovery Tissue	Tumor tissue sample
MG22	GIST	BGI SENTIS Cancer+Discovery ctDNA	Liquid biopsy sample
MG23	Pancreatic cancer	BGI SENTIS Cancer+Discovery Tissue	Tumor tissue sample
MG24	Lung cancer	BGI SENTIS Cancer+Discovery ctDNA	Liquid biopsy sample
MG25	Colorectal cancer	BGI SENTIS Cancer+Discovery Tissue	Tumor tissue sample
MG26	Sarcoma	FoundationOne Heme	Tumor tissue sample

MG27	small bowel cancer	BGI SENTIS Cancer+Discovery Tissue	Tumor tissue sample
MG28	Colorectal cancer	BGI SENTIS Cancer+Discovery Tissue	Tumor tissue sample
MG29	Pancreatic cancer	BGI SENTIS Cancer+Discovery ctDNA	Liquid biopsy sample
MG30	small bowel cancer	BGI SENTIS Cancer+Discovery Tissue	Tumor tissue sample
MG31	Ovarian cancer	BGI SENTIS Cancer+Discovery Tissue	Tumor tissue sample
MG32	Breast cancer	BGI SENTIS Cancer+Discovery Tissue	Tumor tissue sample
MG33	Colorectal cancer	BGI SENTIS Cancer+Discovery Tissue	Tumor tissue sample
MG34	Gastric cancer	FoundationOne CDx	Tumor tissue sample
MG35	Breast cancer	FoundationOne CDx	Tumor tissue sample
MG36	Gastric cancer	MD Anderson Madrid	Tumor tissue sample
MG37	Appendix cancer	Caris Comprehensive Tumor Profiling	Tumor tissue sample
MG38	Ovarian cancer	Caris Comprehensive Tumor Profiling	Tumor tissue sample
MG39	Ovarian cancer	Caris Comprehensive Tumor Profiling	Tumor tissue sample
MG40	Pancreatic cancer	Admera oncoGxone	Tumor tissue sample
MG41	Breast cancer	Caris Comprehensive Tumor Profiling	Tumor tissue sample
MG42	Sarcoma	OncoDeep	Tumor tissue sample
MG43	Pancreatic cancer	BGI SENTIS Cancer+Discovery Tissue	Tumor tissue sample
MG44	Colorectal cancer	FoundationOne liquid	Liquid biopsy sample
MG45	Pancreatic cancer	Caris Comprehensive Tumor Profiling	Tumor tissue sample
MG46	Sarcoma	Caris Comprehensive Tumor Profiling	Tumor tissue sample
