# Supplementary material for: Mutational Landscape and Actionable Target Rates on Advanced Stage Refractory Cancer Patients: A Multicenter Chilean Experience
Source: J Pers Med. 2022 Jan 31;12(2):195. doi: 10.3390/jpm12020195 (PMC8879850; doi:10.3390/jpm12020195)
Supplement: Supplementary file 1 [file jpm-12-00195-s001.zip › Supplementary Table S3.pdf]

Supplementary Table S3. Clinical characteristics and main outcomes of selected patients on targeted therapy based on molecular testing.

ID	Sex	Age	Cancer type	Stage	Genes with alterations	FDA approved therapy (in cancer type)	FDA approved therapy (in other cancer type)	Other biomarkers	Previous lines of therapy	Time from diagnosis to molecular testing (months)	Targeted therapy used	Time targeted therapy (months)	Best response
M7	Male	62	Colorectal cancer	IV	*BRAF, CCND2, TP53, FGF2, FGF6, SMAD4, HCK, MECOM , ACVR2A , HGF , FANCA , FAT3 , PTPRD*	No	BRAF inhibitors + anti EGFR or MEK inhibitors	MSS, KRAS codon 59 mut, BRAF 600 codon mut (PCR)	2	10	Regorafenib	3.3	Progressive disease
M11	Female	77	Melanoma	IV	*NRAS, ASXL1 , APC , NTRK3 , SLIT2 , FGFR2 , NCOR1, PBRM1, ATRX , TSHR, MECOM, BRCA2 , SMARCA1, PAX8, PIK3C2A, EPPK1*	Immunotherapy	No	PDL1+ (>1%)	2	16	Nivolumab-Ipilimumab	2.7	Stable disease
M14	Female	33	GIST	IV	*KIT, ATM*	Imatinib	No	KIT wt, PDGFRA wt (PCR)	1	9	Imatinib	26.3	Complete response
M38	Female	88	Ovarian cancer	IV	*BRCA2, STK11, TP53*	PARP inhibitor	No	No	4	37	Olaparib	5.8	Partial response

GIST gastrointestinal stromal tumor, PCR polymerase chain reaction-based assay, wt wild-type, mut mutated, MSS microsatellite stability.
