# Supplementary material for: Mutational Landscape and Actionable Target Rates on Advanced Stage Refractory Cancer Patients: A Multicenter Chilean Experience
Source: J Pers Med. 2022 Jan 31;12(2):195. doi: 10.3390/jpm12020195 (PMC8879850; doi:10.3390/jpm12020195)
Supplement: Supplementary file 1 [file jpm-12-00195-s001.zip › Supplementary Table S4.pdf]

Supplementary Table S4. Clinical characteristics of selected patients without targeted therapy based on molecular testing.

ID	Sex	Age	Cancer type	Clinical stage	Genes with alterations	FDA approved therapy (in cancer type)	FDA approved therapy (in other cancer type)	Other biomarkers	Previous lines of therapy	Time from diagnosis to molecular testing (months)
MG1	Male	60	Pancreatic cancer	IV	*BRCA2, KRAS, CDKN2A*	No	PARP inhibitor	MSS	3	44
MG2	Female	36	Pancreatic cancer	IV	*NTRK2*	No	No	No	2	14
MG3	Male	63	Colorectal cancer	IV	*NF1, FBXW7, APC, ASXL1, TP53*	No	Binimetinib, Cobimetinib, Trametinib	KRAS/NRAS wt (PCR)	5	92
MG4	Male	54	Pancreatic cancer	IV	*KRAS, ARID1A, TP53*	No	No	No	1	6
MG8	Male	76	Gallbladder and bile duct cancer	IV	*ATM, KRAS, TP53, TGFBR2, ABL2, KMT2D, IRS2, RCF1*	No	No	No	1	9
MG10	Female	38	Colorectal cancer	Recurrence	*NOTCH4*	No	No	MSS	1	4
MG12	Male	61	Colorectal cancer	IV	*PML*	No	No	MSS, KRAS wt (PCR)	10	76
MG13	Female	55	Neuroendocrine tumor	IV	*TP53, KIT*	No	No	MSS	2	14
MG15	Female	24	Adrenal cancer	IV	*RB1 , MEN1*	No	No	No	2	17
MG16	Female	37	Adrenal cancer	Recurrence	*HER2, MUTYH*	No	No	No	4	257
MG17	Female	67	Colorectal cancer	IV	*APC, MAP2K4, TP53, DNMT3A, BRAF, PIK3R1*	No	No	MSS, KRAS/NRAS wt (PCR)	3	15
MG19	Male	63	Prostate cancer	IV	*MTOR, KMT2D*	No	No	FISH Ewing Sarcoma Negative, PDL1 Negative	6	34
MG20	Male	84	Pancreatic cancer	IV	*CDK6, MTAP, TP53, U2AF1*	No	No	No	0	1
MG22	Male	74	Gastrointestinal stromal tumor	IV	*KIT, DNMT3A*	Imatinib	No	KIT exon 11 mut (Sanger seq)	4	63

MG23	Male	72	Pancreatic cancer	IV	*KRAS, TP53*	No	No	MSI (MSH6 absence)	3	28
MG24	Female	80	Lung cancer	IV	*TRRAP, MUTYH*	No	No	PDL1+, ALK Negative, EGFR wt (PCR)	2	32
MG25	Female	40	Colorectal cancer	IV	*KRAS, TP53, APC, ERBB4, MSH6, HGF, WISP3, SLIT2, EXT1*	Regorafenib	No	MSI, KRAS/NRAS/BRAF wt (PCR)	4	75
MG26	Female	77	Sarcoma	IV	*NTRK3, CDKN2A, PRDM1*	Larotrectinib	No	FISH synovial sarcoma Negative	2	8
MG28	Male	51	Colorectal cancer	IV	*MUTYH, TP53, RBM10, APC*	No	No	MSS, KRAS codon 12 mut, NRAS/BRAF wt	5	19
MG29	Male	58	Pancreatic cancer	IV	*No alterations found*	No	No	No	2	3
MG30	Male	52	small bowel cancer	IV	*TP53, KRAS, FGFR3, SMAD4, APC, SOX9, FGF3, FGF4, IGF2, HDAC1*	No	No	No	3	2
MG31	Female	68	Ovarian cancer	IV	*AKT2, MET, ERBB2, ARID1A, MCL1*	No	Everolimus	No	3	17
MG32	Female	43	Breast cancer	IV	*CCND1, ESR1, BTG1, FGF19, FGF3, FGF4, ETV6*	Ribociclib	No	No	7	91
MG33	Female	32	Colorectal cancer	IV	*BRAF, PIK3CA, PTEN, ACVR2A, CDKN1B, HDAC4, PTP4A3, APC, TP53, CHD2, KDR, RPS6KB1, BMPR1A, NKX3-1, NCOA2*	No	BRAF inhibitors + anti EGFR or MEK inhibitors	KRAS/NRAS wt, BRAF mut (PCR)	3	7
MG36	Female	47	Gastric cancer	IV	*ESR1_CCDC170, ARID1A, PIK3CA, ERBB2*	No	No	HER2+	3	6

MG37	Male	53	Appendix cancer	IV	*KRAS, MED12*	No	No	No	4	38
MG40	Female	69	Pancreatic cancer	IV	*KDM6A*	No	No	No	2	4
MG42	Female	52	Sarcoma	IV	*TP53*	No	No	No	1	15
MG43	Male	46	Pancreatic cancer	IV	*IDH2*	No	No	No	4	21
MG44	Female	61	Colorectal cancer	IV	*KRAS*	No	No	MSS, KRAS codon 12 mut, NRAS/BRAF wt	5	29
MG46	Female	71	Sarcoma	Localizad	*MDM2, CDK4, EP300*	No	No	No	2	109

PCR polymerase chain reaction-based assay, wt wild-type, mut mutated, MSS microsatellite stability, MSI microsatellite instability, seq sequencing.
