# Supplementary figures and images for: Mutational Landscape and Actionable Target Rates on Advanced Stage Refractory Cancer Patients: A Multicenter Chilean Experience
Source: J Pers Med. 2022 Jan 31;12(2):195. doi: 10.3390/jpm12020195 (PMC8879850; doi:10.3390/jpm12020195)

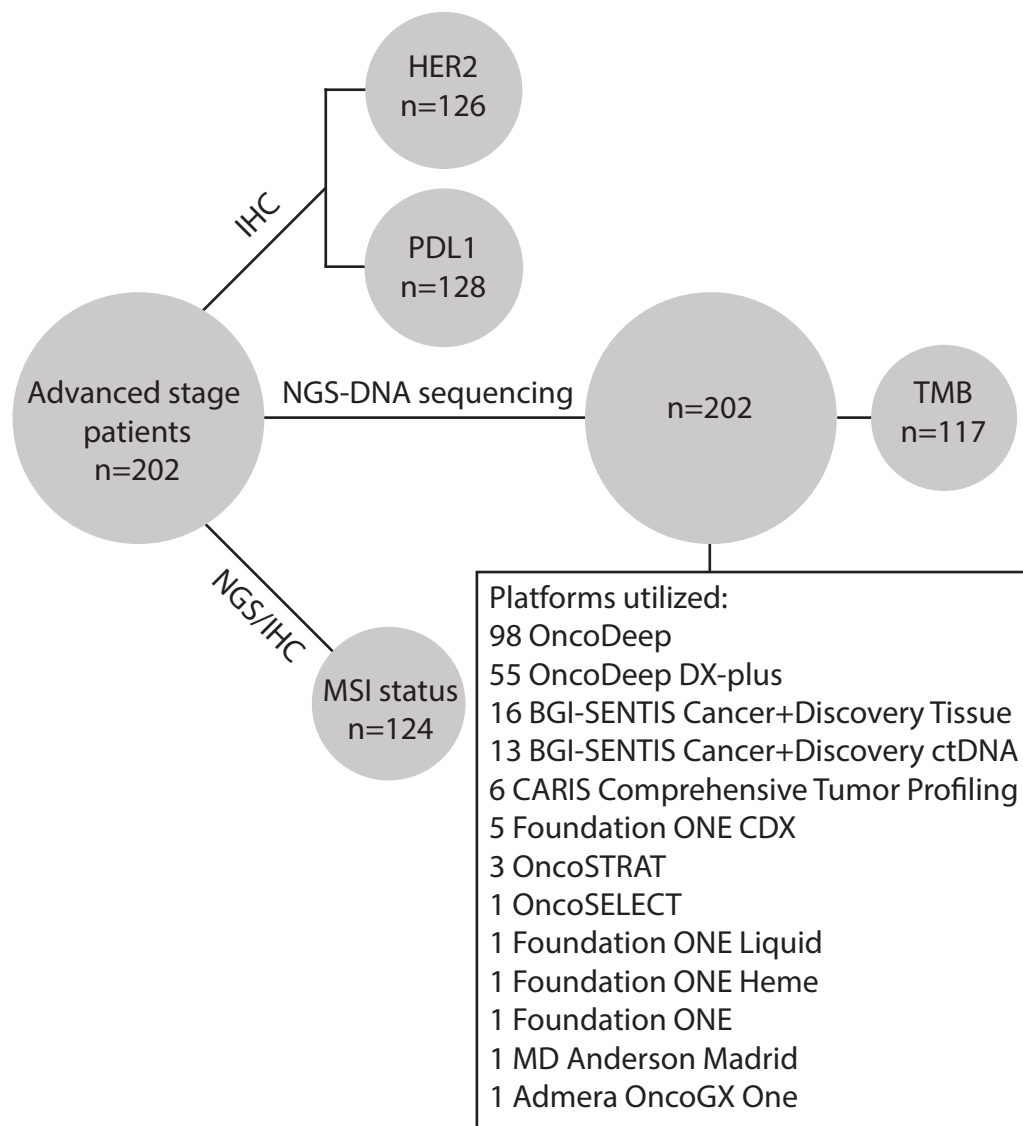

Supplement: Supplementary file 1 [file jpm-12-00195-s001.zip › Supplementary FigS1 copia.pdf]
